# Gut Microbiota as Potential Therapeutic Target for the Treatment of Cow’s Milk Allergy

**DOI:** 10.3390/nu5030651

**Published:** 2013-03-01

**Authors:** Roberto Berni Canani, Margherita Di Costanzo

**Affiliations:** 1 Food Allergy Unit, Department of Translational Medicine, Pediatric Section, University of Naples “Federico II”, Naples 80131, Italy; E-Mail: mara.dicostanzo@live.it; 2 European Laboratory for the Investigation of Food Induced Diseases, University of Naples “Federico II”, Naples 80131, Italy

**Keywords:** food allergy, probiotics, intestinal microflora, immune system, tolerance acquisition

## Abstract

Cow’s milk allergy (CMA) continues to be a growing health concern for infants living in Western countries. The long-term prognosis for the majority of affected infants is good, with about 80% naturally acquiring tolerance by the age of four years. However, recent studies suggest that the natural history of CMA is changing, with an increasing persistence until later ages. The pathogenesis of CMA, as well as oral tolerance, is complex and not completely known, although numerous studies implicate gut-associated immunity and enteric microflora, and it has been suggested that an altered composition of intestinal microflora results in an unbalanced local and systemic immune response to food allergens. In addition, there are qualitative and quantitative differences in the composition of gut microbiota between patients affected by CMA and healthy infants. These findings prompt the concept that specific beneficial bacteria from the human intestinal microflora, designated probiotics, could restore intestinal homeostasis and prevent or alleviate allergy, at least in part by interacting with the intestinal immune cells. The aim of this paper is to review what is currently known about the use of probiotics as dietary supplements in CMA.

## 1. Introduction

During the last decade, we observed a changing pattern in cow’s milk allergy (CMA), the most common food allergy in childhood. An increased prevalence, severity of clinical manifestations and risk of persistence was demonstrated in Western countries [1]. In Italy, CMA is responsible for 42% of food-induced anaphylaxis in the pediatric population [2]. 

Much evidence indicates the development of intestinal microflora as a crucial factor for immune system maturation and tolerance acquisition [3]. Early epidemiological studies supported the idea that environment-induced alterations in the composition of intestinal microflora play a central role in the development of allergic diseases [4]. A recently developed ultra-high-throughput sequencer, called a pyrosequencer, allowed sequence-based 16S rRNA profiling of microbiota, confirming the presence of gut dysbiosis in allergic infants. In particular, a decrease in selected *Firmicutes* species and an increase in *Bacteroidetes* species was demonstrated [5]. For more than a century, probiotics have been used as a therapeutic/preventive strategy for a variety of gastrointestinal disorders, restoring the intestinal microflora. The World Health Organization (WHO)/Food and Agriculture Organization of the United Nations (FAO) define probiotics as live microorganisms that, when consumed in adequate amounts as part of food or as oral supplements, confer a health benefit on the host [6]. Probiotics research and the industry have continued to grow from these early observations, and the global sales of probiotic ingredients are expected to reach $31.1 billion by 2015, with an annual growth rate of 7.6% for the next few years [7]. Despite the plethora of basic research data, probiotic clinical research in food allergy is still in its infancy, but the most recent evidence supports the potential clinical impact derived from a manipulation of intestinal microflora as a disrupting strategy to efficiently address the changing pattern of CMA.

## 2. Oral Tolerance and Intestinal Microflora

Food antigens and intestinal microflora constitute the majority of the antigen load in the intestine, and the “default” reaction of the immune system confronted with them leads to systemic unresponsiveness. This phenomenon is known as oral tolerance and is a key feature of intestinal immunity [8]. The complex interaction between intestinal contents and immune and non-immune cells results in an environment that favors tolerance by the induction of IgA antibodies and CD4^+^ T regulatory cells (producing IL-10 and IFN-γ) [3]. This ensures that a homeostatic balance is maintained between the intestinal immune system and its antigen load, so that it retains the ability to recognize dangerous and harmless antigens as foreign and preserves the integrity of the intestinal mucosa. 

**Figure 1:**
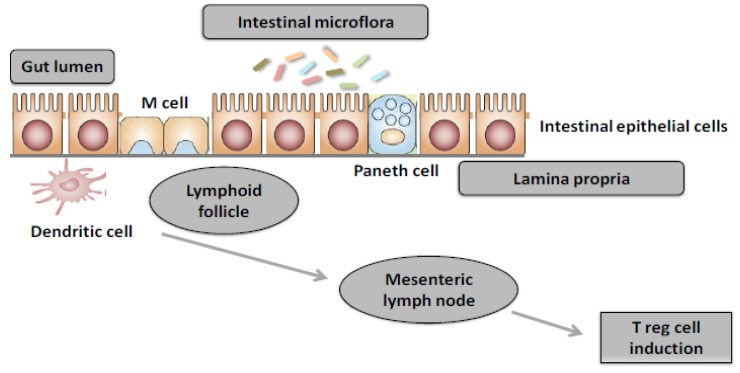
Intestinal microflora drives oral tolerance development. Under homeostatic condition, antigens from selected components of intestinal microflora are acquired in the lamina propria and presented in the mesenteric lymphonodes by CD 103^+ ^dendritic cells. Through mechanisms mainly involving transforming growth factor (TGF) β and retinoic acid, dendritic cells induce the production of gut homing Treg cells. Treg cells actively suppress allergic sensitization to food.

The inappropriate immune response to food, which is responsible for food allergy, is the result of a deregulation of these crucial processes [9]. An allergic reaction mainly corresponds to the activation of Th2 cells against food allergens and occurs in two phases: the first phase corresponds to transport of the allergen through the intestinal barrier, its capture by antigen presenting cells, dendritic cells (DCs) or enterocytes, and its presentation to naive Th0 cells, which differentiate in the presence of IL-4 into Th2 cells. Activated Th2 cells then produce an IL-4 cytokine that enables the production of allergen-specific IgE by B-cells [10]. These secreted IgEs then bind to mast cells via the IgE receptor, FcεRI. The activation phase corresponds to the degranulation of mast cells after further exposure to the same allergen that links directly with specific IgE on the surface of these cells. This phenomenon triggers release of the allergic mediators involved in clinical manifestations of allergy. Recent data strongly suggest that gut microbiota is important for oral tolerance development [11] (see Figure 1).

Basic research involving microbiology, biology, immunology and genetics is providing interesting insights on the delicate network driving to oral tolerance. Studies on germ-free mice revealed a failure in the acquisition of tolerance to food proteins. Mice with food allergy exhibit a specific gut microbiota signature capable of transmitting disease susceptibility. Transplanted healthy infant microbiota had a protective impact on sensitization and CMA in mice. Finally, polymorphisms in or deficiency of microbial sensors for bacterial lipopolysaccharide (LPS) (TLR-4) are associated to food allergy [12,13]. The spore-forming component of indigenous intestinal microbiota, particularly clusters IV and XIVa of the genus *Clostridium*, promote Treg cell accumulation. Colonization of mice by a defined mix of *Clostridium* strains provides an environment rich in TGF β and affected Foxp3+ Treg number and function in the colon. Oral inoculation of *Clostridium* during the early life of conventionally reared mice results in resistance to allergic colitis and systemic immunoglobulin E responses in adult mice, suggesting a new therapeutic approach to food allergy [14]. In this light, it is important to consider that after four weeks of treatment with the probiotic* Lactobacillus rhamnosus* GG (LGG), it is possible to induce a significant increase in clostridia in milk-hypersensitive subjects [15].

## 3. Probiotics and Their Mechanisms of Action

Probiotics have pleiotropic effects that occur within the intestinal lumen or within and beyond the intestinal mucosa (Table 1). Local influences of probiotics include: hydrolysis of antigenic peptides in the gut lumen, modulation of intestinal permeability and reduction of systemic penetration of antigens, increased local IgA production and modulation of local inflammation and stimulation of epithelial cell growth and differentiation [16,17,18,19]. Some systemic activities consist of anti-inflammatory effects mediated by toll-like receptors (TLRs), Th1 skewing of responses to allergens and activation of tolerogenic DCs, in addition to T regulatory cell production and tolerance acquisition [20,21].

**Table 1:** Schematic representation of the mechanisms of action of probiotics implicated in allergy prevention and treatment.

	Effects
Within intestinal lumen	➢ Modulation of intestinal microflora [16]
	➢ Increased local IgA production [17]
	➢ Hydrolysis of antigenic peptides [18]
At mucosal level	➢ Modulation of intestinal permeability [19]
	➢ Stimulation of cell growth and differentiation [20]
Beyond the intestinal mucosa	➢ Modulation of innate/adaptive immune system [3]
	➢ Induction of oral tolerance [3]
	➢ Impact on the enteric nervous system [21]

It is becoming evident that completely different effects may be observed, depending on the species and the strain of the microorganism used [22]. Recent *in vivo* studies in healthy human volunteers measured the changes in gene transcription profiles to determine the molecular responses that occur in the human duodenal mucosa following consumption of probiotic *Lactobacillus* spp. [23,24]. These nutrigenomic studies showed that the mucosal responses to distinct *Lactobacilli* are profoundly different, illustrating the specificity of the host responses to specific bacterial strains and/or species [24] or even different preparations of the same bacterial strain [23]. Many effects elicited by probiotics are dependent on epigenetic modulation of gene expression [25]. These effects could be important during critical periods of early development, for example, in the development and programming of immune tolerance in the newborn [26].

## 4. Animal Models

Numerous animal and human studies have been performed to test the potential effects of various strains of probiotic bacteria. In this context, one of the most extensively studied probiotics worldwide is LGG. Preventive and therapeutic properties of LGG related to atopic diseases, particularly in infants with CMA, have been reported [27]. Animal models for food allergy provide an interesting tool to perform mechanistic research and to investigate the safety and efficacy of new therapeutic and preventive approaches for food allergy. Much progress has been made in recent years in developing an animal model of CMA. In particular, animal models for CMA using oral sensitization are mimicking the human situation, as children are most likely sensitized to cow’s milk via the oral route. Oral tolerance to cow’s milk proteins has been studied in these models aiming to prevent both systemic and mucosal responses. In BALB/c mice that were sensitized with cow’s milk proteins via the systemic route, oral LGG supplementation favorably modulated immune reactions by shifting Th2-dominated trends toward Th1-dominated responses [28]. 

## 5. Human Studies

### 5.1. Prevention of CMA

Most randomized controlled trials enrolled infants at high risk for developing allergy, which was defined as more than one family member having any allergic disease. Most of these studies looked primarily at early outcomes of allergic disease, such as eczema. Although atopic eczema is a frequent manifestation of CMA [29], it is hard to define a selective preventive effect against this type of food allergy. A large number of papers have been published on this topic with conflicting results. Differences in study design, populations, probiotic strains and dosages are responsible for these discrepancies. Prenatal and postnatal administration of high doses of selected probiotic strains seems to be the most promising approach (see Table 2). 

**Table 2:** Main allergy prevention studies using probiotics.

Investigators	Population	Probiotics and doses	Prenatal administration	Postnatal administration	Reduction in eczema	References
Kalliomaki *et al.* (2001, 2002, 2003)	Mothers with ≥1 first-degree relative (or partner) with allergic disease	*Lactobacillus rhamnosus* GG (1 × 10^10^ CFU/day) (only to mother if breast feeding post-natally)	*Yes*	*Yes*	*Yes*	[30,31,32]
			2–4 weeks before delivery	6 months (only to baby if not breastfeeding)	at 2 and 4 years	
Rautava *et al.* (2006)	Need for artificial feeding before 2 months of age	*Lactobacillus rhamnosus* GG (1 × 10^10 ^CFU/day) + *Bifidobacterium lactis* (1 × 10^10 ^CFU/ day) added to infant formula	*No*	*Yes*	*No*	[33]
				from <2 months (depending on age started formula) until 12 months		
Taylor *et al.* (2007)	Mother with positive SPT or documented allergic disease	*Lactobacillus acidophilus* (3 × 10^8^ CFU/day)	*No*	*Yes*	*No*	[34]
				6 months direct to infant	at 1 year	
Kukkonen *et al.* (2007, 2009)	One or both parents with allergic disease	*Lactobacillus rhamnosus* GG and LC705 (both 5 × 10^9^ CFU twice daily) + *Bifidobacterium breve* and *Proprionibacterium freudenreichii* (both 2 × 10^9^ CFU twice daily)	*Yes*	*Yes*	*Yes*	[35,36]
			2–4 weeks before delivery	6 months direct to infant	At 2 years. No effect at 5 years (except decrease in atopic eczema in cesarean-delivered children)	
Abrahamsson *et al.* (2007)	Families with allergic disease	*Lactobacillus reuteri* (1 × 10^8^ CFU/day)	*Yes*	*Yes*	*No*	[37]
			2–4 weeks before delivery	12 monthsdirect to infant	At 2 years	
Kopp *et al.* (2007)	Pregnant women from families with ≥1 first-degree relative with an atopic disease	*Lactobacillus rhamnosus* GG (1 × 10^10^ CFU/day) to mother if breast feeding post-natal for 3 months, then to the neonates for additional 3 months	*Yes*	*Yes*	*No*	[38]
			4–6 weeks before delivery	6 monthsdirect to infant	At 2 years	
Wickens *et al.* (2008)	One or both parents with allergic disease	*Lactobacillus rhamnosus* HN001 (1 × 10^10^ CFU/day) or *Bifidobacterium lactis* (1 × 10^10^ CFU/day) HN019	*Yes*	*Yes*	*Yes*	[39]
			2–5 weeks before delivery	2 years to infant, regardless of feeding method	at 2 years	
Huurre *et al.* (2008)	Mother with current atopic disease	*Lactobacillus rhamnosus* GG + *Bifidobacterium lactis* (both at 1 × 10^10^ CFU/day)	*Yes*	*Yes*	*No*	[40]
			from first trimester	end of exclusive breastfeeding		
Soh *et al.* (2009)	Any first degree relative with SPT + allergic disease	*Lactobacillus rhamnosus* LPR (1 × 10^9^ CFU/day) + *Bifidobacterium longum* BL999 (6 × 10^8^ CFU/day)	*No*	*Yes*	*No*	[41]
				6 monthsin infant formula	at 1 year	
Niers *et al.* (2009)	Atopic disease in either mother or father plus at least one sibling	*Lactococcus lactis W58 + Bifidobacterium lactis W52 + Bifidobacterium bifidum W23* (each at: 1 × 10^9^ CFU/day)	*Yes*	*Yes*	*Yes*	[42]
			6 weeks beforedelivery	12 months (direct to infant)		
West *et al.* (2009)	Atopic disease in either mother or sibling	*Lactobacillus paracasei* strain F19 (1 × 10^8^ CFU/day in weaning cereal)	*No*	*Yes*	*Yes*	[43]
				4–13 months during weaning		
Dotterud *et al.* (2009)	Unselected population	*Lactobacillus rhamnosus* GG + *Lactobacillus acidophilus* LA5 +* Bifidobacterium lactis* Bb-12 (each at 5 × 10^10^ CFU/day)	*Yes*	*No*	*Yes*	[44]
			from 36 weeks	Given to the breastfeeding mother for 3 months		
Kim *et al.* (2010)	Pregnant women with a family history of allergic diseases	*Bifidobacterium bifidum* BGN4 + *Bifidobacterium lactis* AD011 and *Lactobacillus acidophilus* AD031 (each at 1.6 × 10^9^ CFU/day) in 0.72 g of maltodextrin and 0.8 g of alpha-corn	*Yes*	*Yes*	*Yes*	[45]
			4–8 weeks before delivery	6 months after delivery	at 1 year	
Boyle *et al.* (2011)	Pregnant women carrying infants at high risk of allergic disease	*Lactobacillus rhamnosus* GG (1.8 × 10^10^ CFU/day)	*Yes*	*No*	*No*	[46]
			from 36 weeks gestation until delivery		at 1 year	
Rautava *et al.* (2012)	Mothers with allergic disease and atopic sensitization	*Lactobacillus rhamnosus* LPR + *Bifidobacterium longum* BL999 or* Lactobacillus paracasei* ST11 + *Bifidobacterium longum* BL999 (each at 1 × 10^9^ CFU/day)	*Yes*	*Yes*	*Yes*	[47]
			2 months before delivery	2 months of breast feeding		

SPT: skin prick test; CFU: colony-forming unit.

### 5.2. Treatment of CMA

The first objective in the treatment of CMA is the rapid resolution of symptoms. At this time, the only proven treatment consists of elimination of cow’s milk protein from the diet. For infants receiving standard formulas, a hypoallergenic formula is indicated. Administration of LGG to food-allergic children (age <2 years, challenge-proven and mild-to-moderate eczema) improved the eczema score significantly [48]. Studies in infants with eczema who received formulas supplemented with LGG showed benefits in decreasing gastrointestinal symptoms [49]. For instance, after a challenge study in infants allergic to cow’s milk proteins, fecal IgA levels were detected to be higher, and TNF-α levels were lower in the LGG applied group compared to the placebo [50]. Nermes *et al*. [51] investigated the interaction of LGG with skin and intestinal microflora and humoral immunity in infants with atopic dermatitis. This study showed a statistically significant decrease of IgA- and IgM-secreting cells one month after starting an intervention with extensively hydrolyzed casein formula (eHCF) supplemented with LGG. This might indirectly indicate that LGG enhances gut barrier function and accelerates immunological maturation in infants with atopic dermatitis. Especially, the finding of significant increase in memory B cells in LGG treated infants could be of particular importance [51]. Moreover, LGG is able to induce IFN-γ secretion in infants with CMA and in infants with IgE-associated dermatitis, but not in infants without CMA. This supports the view that the pattern of intestinal microflora may be aberrant in infants with an atopic predisposition, and the beneficial effects of probiotics are evident only in this group [52]. The addition of LGG to an eHCF significantly improved the recovery of the inflamed colonic mucosa if compared to that obtained with eHCF alone in infants with blood in the stool and CMA-induced colitis, as indicated indirectly by greater decreases in fecal calprotectin and in the number of infants with persistence of occult blood in stools after 1 month [53].

The second objective in the treatment of CMA is tolerance acquisition. Hol, J. *et al.* [54] showed that supplementation of a combination of *Lactobacillus casei* CRL431and *Bifidobacterium lactis* Bb-12 to an extensively hydrolyzed formula failed to induce additional or accelerated cow’s milk (CM) tolerance during 12 months of treatment in infants with CMA. In contrast, we recently demonstrated that an eHCF containing LGG was able to accelerate the development of tolerance acquisition in infants affected by CMA. Infants (aged 1–12 months), consecutively referred for strongly suspected CMA, but still receiving cow’s milk proteins, were invited to participate in the study. Subjects were randomly allocated to one of the two groups of dietary interventions: group 1, received an eHCF and group 2 received an eHCF containing LGG (at least 1.4 × 10^7^ CFU/100 mL). After 12 months, the double-blind placebo-controlled food challenge (DBPCFC) was negative in 15 of 28 infants in the control group (53.6%) and in 22 of 27 infants receiving the eHCF containing LGG (81.5%, *p* = 0.027). These findings suggest an innovative approach for infants affected by CMA, namely an “active dietotherapy” able to reduce the time of tolerance acquisition [55].

## 6. Safety

The addition of probiotics in formulas used for the management of CMA requires that they be proven safe and are well tolerated. According to the European Society of Pediatric Gastroenterology and Nutrition (ESPGHAN) and the American Academy of Pediatrics, a formula must be tested in a properly designed DBPCFC and can be considered hypoallergenic when demonstrated with 95% confidence that at least 90% of infants and children with confirmed CMA would have no reaction to the formula under double-blind, placebo-controlled conditions. LGG has over 25 years of safe use, including administration to preterm infants. Recently, Muraro *et al.* [56] demonstrated that an eHCF remains hypoallergenic following the addition of LGG, satisfying both the ESPGHAN and American Academy of Pediatrics guidelines. 

An emerging problem is the observation that some probiotic compounds that are currently on the market may contain hidden allergens of food and may not be safe for subjects with CMA. Thus, more accurate screening tests to detect residual food proteins in end products are necessary to assess the safety of these products for food allergic patients. For allergic subjects, we would only recommend well characterized products with better information on their labels about the content of cow’s milk proteins [57].

## 7. Conclusions

An increasing amount of evidence suggests the role of select probiotics in prevention or treatment of CMA. These data support the importance of a “nutritional immunology approach” able not only to efficiently cure the symptoms, but also to accelerate tolerance acquisition in children with CMA. However, as a result of strain, dose and product specificities and in order to be in agreement with recommendations of official and scientific organizations, it is important that randomized, controlled trials are performed for each commercialized product. 
